# Molecular phylogenetics and biogeography of the mint tribe Elsholtzieae (Nepetoideae, Lamiaceae), with an emphasis on its diversification in East Asia

**DOI:** 10.1038/s41598-017-02157-6

**Published:** 2017-05-17

**Authors:** Pan Li, Zhe-Chen Qi, Lu-Xian Liu, Tetsuo Ohi-Toma, Joongku Lee, Tsung-Hsin Hsieh, Cheng-Xin Fu, Kenneth M. Cameron, Ying-Xiong Qiu

**Affiliations:** 1 Key Laboratory of Conservation Biology for Endangered Wildlife of the Ministry of Education, and Laboratory of Systematic & Evolutionary Botany and Biodiversity, College of Life Sciences, Zhejiang University, Hangzhou, 310058 P.R. China; 2 College of Life Sciences, Zhejiang Sci-Tech University, Hangzhou, 310018 P.R. China; 3 Botanical Gardens, Graduate School of Science, The University of Tokyo, Tokyo, 112-0001 Japan; 4 Department of Environment and Forest Resources, Chungnam National University, Daejeon, 34134 South Korea; 5 Department of Ecoscience and Ecotechnology, National University of Tainan, Tainan, 700 Republic of China; 6 Department of Botany, University of Wisconsin, Madison, Wisconsin 53706 USA

## Abstract

*Elsholtzia* and its allied genera such as *Collinsonia* and *Perilla* (tribe Elsholtzieae, Lamiaceae) are an ecologically and economically important plant group consisting of ~71 species, with most species distributed in East and Southeast Asia, and several species in North America. Their phylogeny and historical biogeography resulting in a distant intercontinental disjunction are poorly understood. Here we use two nuclear (ETS, ITS) and five chloroplast (*rbcL*, *matK*, *trnL-F*, *ycf1*, *ycf1-rps15*) fragments to reconstruct the phylogeny, biogeographic history, and patterns of diversification of Elsholtzieae. The tribe Elsholtzieae is monophyletic and divided into five clades. The woody *Elsholtzia* species are nested within herbaceous ones and are inferred to have evolved from herbaceous ancestors. Molecular dating shows that the five major clades were established during the Eocene period, but most of the modern diversity did not originate until the Miocene. The divergence between the New World *Collinsonia* and the Old World *Mosla*-*Keiskea*-*Perilla* clade was dated to the mid-Miocene. Ancestral area reconstructions suggest that the tribe originated in East Asia, and then dispersed to Southeast Asia and North America. Overall, our findings highlight the important roles of the uplifts of the Qinghai-Tibetan Plateau (QTP) and climate changes from Middle Miocene onwards in promoting species diversification of Elsholtzieae.

## Introduction

Lamiaceae (the mint family) is the sixth largest family of flowering plants. It is also one of the most economically important families due to the aromatic qualities of most members^1^. Lamiaceae has traditionally been considered closely related to Verbenaceae, but recent phylogenetic studies show that many genera classified in Verbenaceae should be transferred to Lamiaceae^2–4^. This expanded concept of the family contains about 236 genera and over 7,000 species^1^. These have been divided recently into ten subfamilies and two unassigned genera in a large-scale higher level phylogenetic analysis of Lamiaceae^4^.

Among the mint subfamilies, Nepetoideae is the largest, with clearly defined diagnostic morphological characters (e.g., hexacolpate and three-nucleate pollen, an investing embryo, presence of rosmarinic acid). It contains almost half of the genera (~105/236) and about half of the species (~3,600/7,173) in the family^1^. Nepetoideae is recovered as monophyletic in various molecular analyses^4–8^, and is divided into three tribes: Elsholtzieae, Mentheae, and Ocimeae^1^. Tribe Mentheae is large and contains well known genera such as *Mentha* (peppermint), *Nepeta* (catnip), *Origanum* (oregano), *Rosmarinus* (rosemary), *Salvia* (sage), and *Thymus* (thyme). Tribe Ocimeae is smaller, but also contains economically important genera such as ornamental *Coleus*, *Lavandula* (lavender), and *Ocimum* (basil). In contrast, tribe Elsholtzieae includes only about 71 species, and thus is the smallest of these three tribes. However, within many Asian cultures the mint genus *Perilla* of tribe Elsholtzieae is especially well known. The seeds, leaves, and oil of *P. frutescens* (‘shiso’, ‘deulkkae’, ‘tía tô’, ‘beefsteak plant’) are used extensively as condiments and spices in Japanese, Korean, and Vietnamese cuisine.

Although Elsholtzieae has been recovered as monophyletic in previous molecular phylogenetic studies, only two to four genera were used to represent the tribe in those studies^5, 9–12^. Even the most recently published large-scale phylogenetic study of Lamiaceae included just five samples (7%) of Elsholtzieae taxa^4^. Only a recent study included a significant level of taxon sampling for the tribe (7/7 genera; 21/71 species)^13^, but the primary objective of that investigation was to place the enigmatic genus *Ombrocharis* Hand.-Mazz. within the family since it previously had been considered *incertae cedis*. The researchers confirmed that *Ombrocharis* is a member of the tribe and documented a sister relationship with *Perillula* Maxim., with this pair sister to all other members Elsholtzieae. However, the restricted taxon sampling within some of the larger genera (e.g., one identified species of *Keiskea*, one of *Mosla*, two of *Collinsonia*, 14 of 43 *Elsholtzia*) limited the ability to test the monophyly of these genera, or to recover the intrageneric relationships adequately. Although the tribe has an unusual distribution throughout tropical, subtropical, and temperate Asia as well as being disjunct in eastern North America, the historical biogeography and rates of evolution have not been examined due to inadequate taxon sampling.

The seven genera within Elsholtzieae are widely distributed across East and Southeast Asia, except *Collinsonia* L., which is a small genus containing four species from eastern North America^14^. *Keiskea* Miq. contains seven species in China and Japan, and is sometimes merged into *Collinsonia* ^1, 15^. However, this taxonomic treatment is not supported by recent molecular phylogenetic studies^9, 13^ or by pollen morphology^16^. The monotypic *Perillula* (*Perillula reptans* Maxim.) is endemic to South Japan, whereas monotypic *Ombrocharis* (*Ombrocharis dulcis* Hand.-Mazz.) is only found in Hunan Province, Central China. *Perilla* L. is another monotypic genus [*Perilla frutescens* (L.) Britton], commonly found and also widely cultivated across much of East Asia and South/Southeast Asia. *Mosla* Buch.-Ham. ex Benth. contains 14 species distributed mainly in East Asia and Southeast Asia^17^. Finally, as the largest genus in Elsholtzieae, *Elsholtzia* Willd. comprises 43 accepted species. The majority of these occur in the mountain ranges of East Asia, centering on the Sino-Himalayan subkingdom (28/43, ~65%, 11 endemics), but extending to the Sino-Japanese subkingdom (20/43, ~47%, 10 endemics) and Southeast/South Asia (18/43, ~42%, 5 endemics), with only one or possibly three species entering the Qinghai-Tibet Plateau, Mongolian Plateau and Central Asia^12, 17–21^. This is a diverse genus, and a few *Elsholtzia* species are characterized by a woody habit (the only ones in the entire tribe that exhibit obvious secondary growth). All the woody species of *Elsholtzia* occur in the Sino-Himalayan subkingdom, but it is unknown if they evolved from a common ancestor. Several molecular phylogenetic studies of unrelated plant groups have shown that woody species have evolved independently from herbaceous ancestors on numerous occasions^22–24^, and so it is very likely that woody *Elsholtzia* may have originated from a herbaceous ancestor(s) as well. Overall, the eastern Asia (EAS) - eastern North America (ENA) disjunction pattern and highly uneven distribution among genera make Elsholtzieae an excellent model for understanding the evolution of intra- and intercontinental disjunction as well as diversification history and its relation to paleoclimate and paleogeography in East Asia and North America.

Here we provide the most comprehensive phylogenetic analysis of the tribe Elsholtzieae to date (including samples from all seven genera and 76% of the species) using sequence data from the internal and external transcribed spacer regions of nuclear ribosomal DNA (nrDNA) (ITS and ETS), three chloroplast (cp) coding (*ycf1*, *rbcL* and *matK*) and two non-coding (*ycf1-rps15* and *trnL-F*) regions, all of which have been widely used in systematics studies of Lamiaceae^10, 11, 13^. With increased taxon and character sampling, our paper intends to: (1) test the monophyly of the four non-monotypic genera of the tribe Elsholtzieae; (2) reconstruct the phylogeny and biogeographic history of the tribe and its constituent genera; and (3) examine the patterns of species diversification of the tribe as related to Northern Hemisphere paleoclimate and paleogeography.

## Results

### Phylogenetic analyses

**Figure 1:**
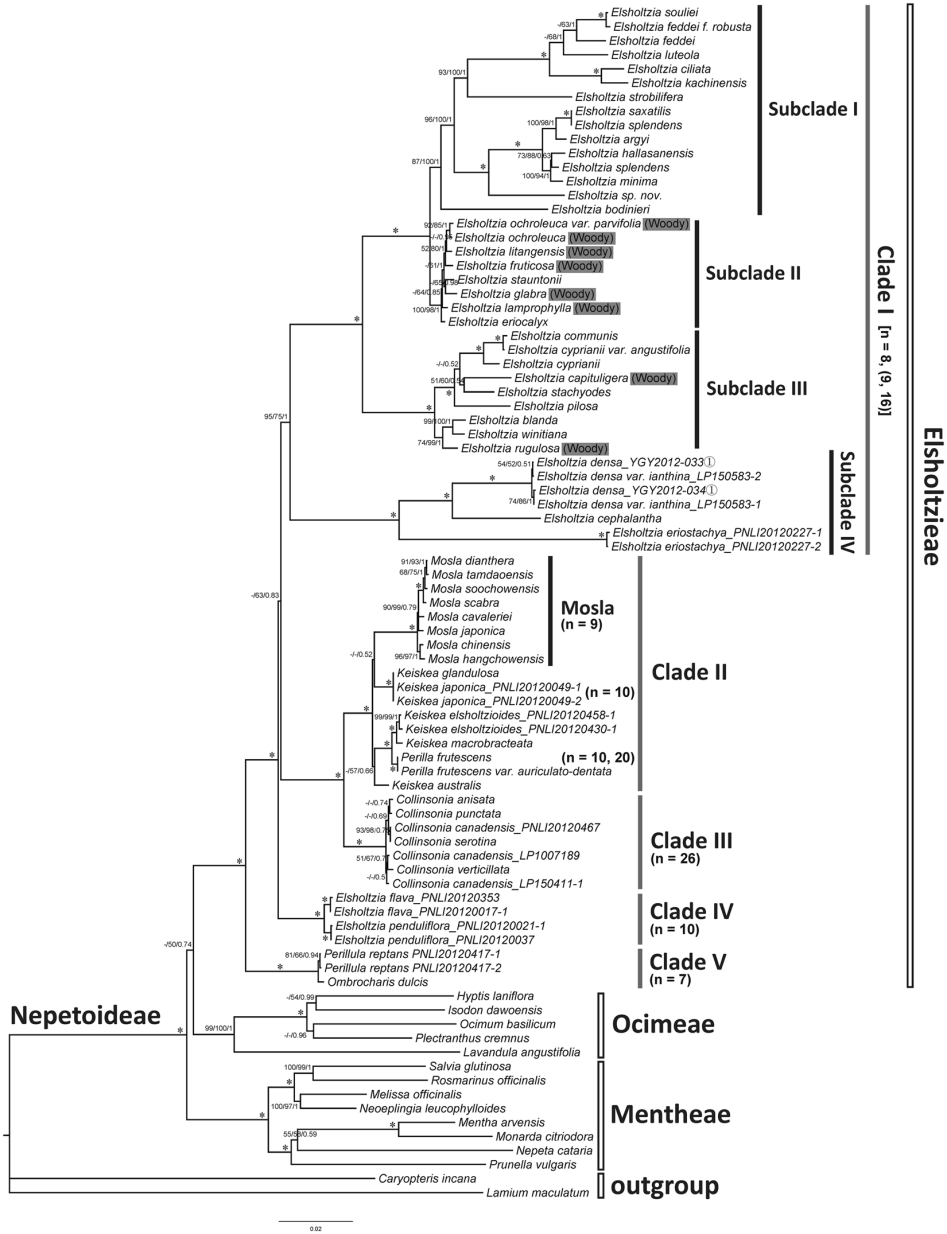
Bayesian 50% majority-rule consensus tree based on the combined cpDNA (*rbcL* + *matK* + *trnL-F + ycf1* + *ycf1-rps15*) + nrDNA (ITS + ETS) dataset, showing the taxa from Elsholtzieae, Ocimeae, and Mentheae, with *Caryopteris incana* and *Lamium maculatum* as the outgroups. The topologies of the maximum parsimony (MP) and maximum likelihood (ML) trees are congruent with the Bayesian inference (BI) tree. MP-BS/ML-BS/BI-PP are shown near corresponding nodes, successively, while “−” indicates support values of less than 50%, and “*” represents full support (100%/100%/1). The known chromosome numbers have been mapped for each clade: Clade I [*n* = 8, (9, 16)]; Clade II (*Mosla*, *n* = 9; *Keiskea japonica*, *n* = 10; *Perilla*, *n* = 10, 20); Clade III (*n* = 26); Clade IV (*n* = 10); Clade V (*Perillula*, *n* = 7).

The tree topologies from MP, ML and Bayesian analyses (with different partitioning strategies) are consistent with each other, and the results based on cpDNA data are similar to those based on combined cpDNA + nrDNA data, but with lower support. Therefore we only present the result of the Bayesian analysis based on our 3-partitioned combined dataset (Fig. 1).

All three tribes are strongly supported as monophyletic (MP-BS ≥ 99%/ML-BS = 100%/BI-PP = 1.00; all values follow this order hereafter). The tribe Elsholtzieae and Ocimeae (*Hyptis*, *Isodon*, *Ocimum*, *Plectranthus* and *Lavandula*) form a weakly supported clade that is sister to the tribe Mentheae (*Monarda*, *Mentha*, *Prunella*, *Nepeta*, *Rosmarinus*, *Salvia*, *Melissa* and *Neoeplingia*). Within Elsholtzieae, *Perillula reptans* and *Ombrocharis dulcis* form a clade (Clade V) with full support (100%/100%/1.00) that is sister to the remainder of the tribe.

The genus *Elsholtzia* is not monophyletic because a strongly supported (*E. flava* + *E. penduliflora*) clade (Clade IV, 100%/100%/1.00) is sister to the rest members of the tribe, although the position of Clade IV in Elsholtzieae is only weakly supported (−/63%/0.83). The remainder of the tribe is divided into two clades, the *Elsholtzia* clade (Clade I, further divided into four subclades: Subclade I–IV) and the *Mosla*-*Keiskea*-*Perilla*-*Collinsonia* clade (Clade II + III). *Elsholtzia densa, E. cephalantha* and *E. eriostachya* form a strongly supported clade (Subclade IV, 100%/100%/1.00) sister to the remaining *Elsholtzia* species (Subclade I + II + III). Except for *E. capituligera* and *E. rugulosa* (two members of Subclade III), all the woody species of *Elsholtzia* appear to have evolved from a recent common ancestor (Subclade II).

Within the *Mosla*-*Keiskea*-*Perilla*-*Collinsonia* clade, the monophyletic *Collinsonia* from North America (Clade III) is sister to the Asian genera (Clade II). The genus *Mosla* is also strongly supported as monophyletic. *Perilla* is clustered with *K. macrobracteata* and *K. elsholtzioides* (100%/100%/1.00), thus rendering *Keiskea* non-monophyletic. However, parts of the Clade II topology are only weakly supported.

The nr topologies were generally similar to the overall phylogeny described above, but with lower support values. There was only one major inconsistency in the nr tree, (*Perillula* + *Ombrocharis*) and (*E. flava* + *E. penduliflora*) form a weak-supported cluster sister to the *Mosla*-*Keiskea*-*Perilla*-*Collinsonia* clade. However, all the visible discordances between the nrDNA and total evidence trees have very low bootstrap or posterior probability values, suggesting there are no hard incongruencies between the datasets.

The highest (14.13–32.20%) and lowest levels of sequence divergence (0–2.99%) were seen in the ETS and *rbcL* sequences, respectively (Table 1). Interspecific sequence variation was found to be 2.5 (ETS) to 9 (*trnL-F*) times higher in *Elsholtzia* species (Clade I) than in the *Mosla*-*Keiskea*-*Perilla* clade (Clade II). In contrast, interspecific sequence variation was lowest in the *Collinsonia* species (Clade III; Table 1).

**Table 1:** Sequence characteristics of *Elsholtzia*, *Mosla*-*Keiskea*-*Perilla*, *Collinsonia*, and sequence divergence values, which were estimated with pairwise distance (p-distance, pairwise deletion).

Characteristics	ITS	ETS	*rbcL*	*matK*	*trnL-F*	*ycf1*	*ycf1-rps15*	chloroplast	combined
No. sequences	62	62	63	59	62	63	56	63	63
Missing data (%)	1.59	1.59	0	6.35	1.59	0	11.11	0	0
Sequence length (bp)	697	441	501	885	935	4278	752	7351	8489
Intra *Elsholtzia* (%)	0–18.59	0–34.29	0–2.99	0–7.88	0–5.98	0–13.30	0–11.72	0.02–10.07	0.01–12.09
Intra *Mosla*-*Keiskea*-*Perilla* (%)	0–3.75	0–13.48	0–0.80	0–1.41	0–0.65	0–1.81	0–1.78	0–1.44	0–2.26
Intra *Collinsonia* (%)	0–0.87	0–1.57	0	0–0.24	0–0.26	0–0.25	0–0.36	0–0.21	0.01–0.37
Among clades (%)	6.03–15.75	14.13–32.20	0–2.99	1.18–6.79	0.39–5.29	1.11–10.98	1.24–10.10	0.81–8.32	2.19–12.15

### Divergence time estimations

**Figure 2:**
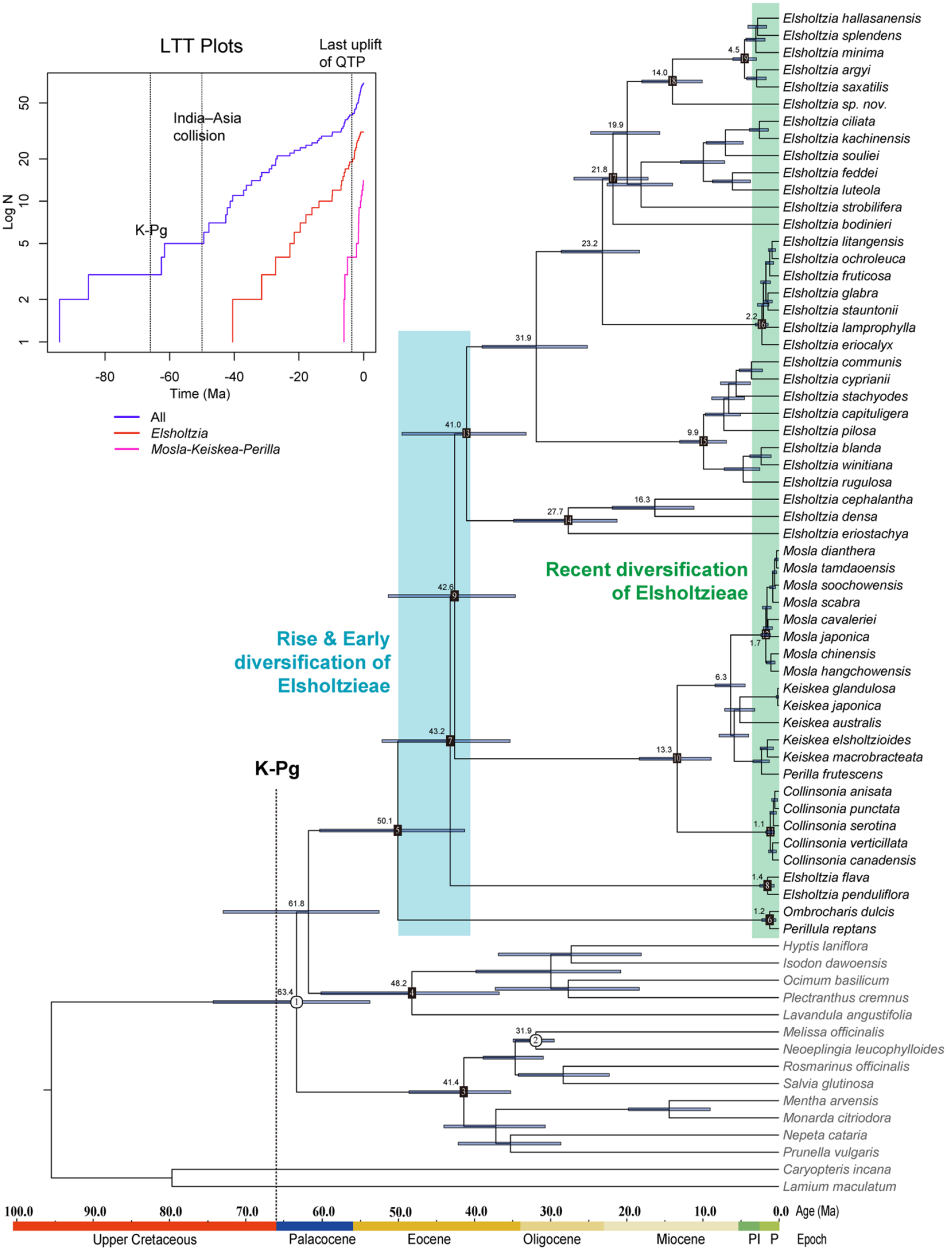
Phylogenetic chronogram of Elsholtzieae as inferred from beast analysis based on two calibration points (node 1 & node 2). Blue bars represent 95% highest posterior density of node age. Our results indicate that an early diversification of Elsholtzieae occurred in Middle Eocene after its rise in Early Eocene (blue shading), and a recent rapid diversification took place after the end of the Miocene (green shading). Standard LTT plots for Nepetoideae, *Elsholtzia*, and the *Mosla-Keiskea-Perilla* clade are presented in the upper left.

The beast chronogram is depicted in Fig. 2. The crown age of Nepetoideae (node 1) was estimated at 63.4 Ma (95% HPD: 53.8–74.4 Ma). The crown group age of the tribe Elsholtzieae (node 5) was estimated to be 50.1 Ma (95% HPD: 41.3–60.3 Ma), while those of the tribes Mentheae (node 3) and Ocimeae (node 4) were estimated at 41.4 Ma (95% HPD: 35.2–48.6 Ma) and 48.2 Ma (95% HPD: 36.8–60.2 Ma), respectively.

The early diversification of an Elsholtzieae ancestor giving rise to modern lineages occurred between 41.0–50.1 Ma (blue shading in Fig. 2). Within Elsholtzieae, contemporary *Perillula* and *Ombrocharis* diverged from each other only around 1.2 Ma (node 6, 95% HPD: 0.3–2.2 Ma). The age of the stem and crown nodes of *E. flava* + *E. penduliflora* clade were estimated at 43.2 Ma (node 7, 95% HPD: 35.3–52.2 Ma) and 1.4 Ma (node 8, 95% HPD: 0.6–2.5 Ma), respectively. The *Mosla*-*Keiskea*-*Perilla*-*Collinsonia* clade arose around 42.6 Ma (node 9, 95% HPD: 34.6–51.3 Ma), and the divergence between the New World *Collinsonia* and the Old World genera was estimated at 13.3 Ma (node 10, 95% HPD: 8.9–18.3 Ma). The genus *Collinsonia* started to diversify around 1.1 Ma (node 11, 95% HPD: 0.6–1.7 Ma), while *Mosla* diversified a little earlier (node 12, 1.7 Ma, 95% HPD: 1.1–2.3 Ma). The primary speciation events within core *Elsholtzia* Subclades I–IV leading to today’s extant taxa started from 27.7 Ma (node 14, 95% HPD: 21.2–34.9 Ma) in the Early Oligocene, but mostly took place after the end of the Miocene (green shading in Fig. 2).

### Ancestral area reconstructions

**Figure 3:**
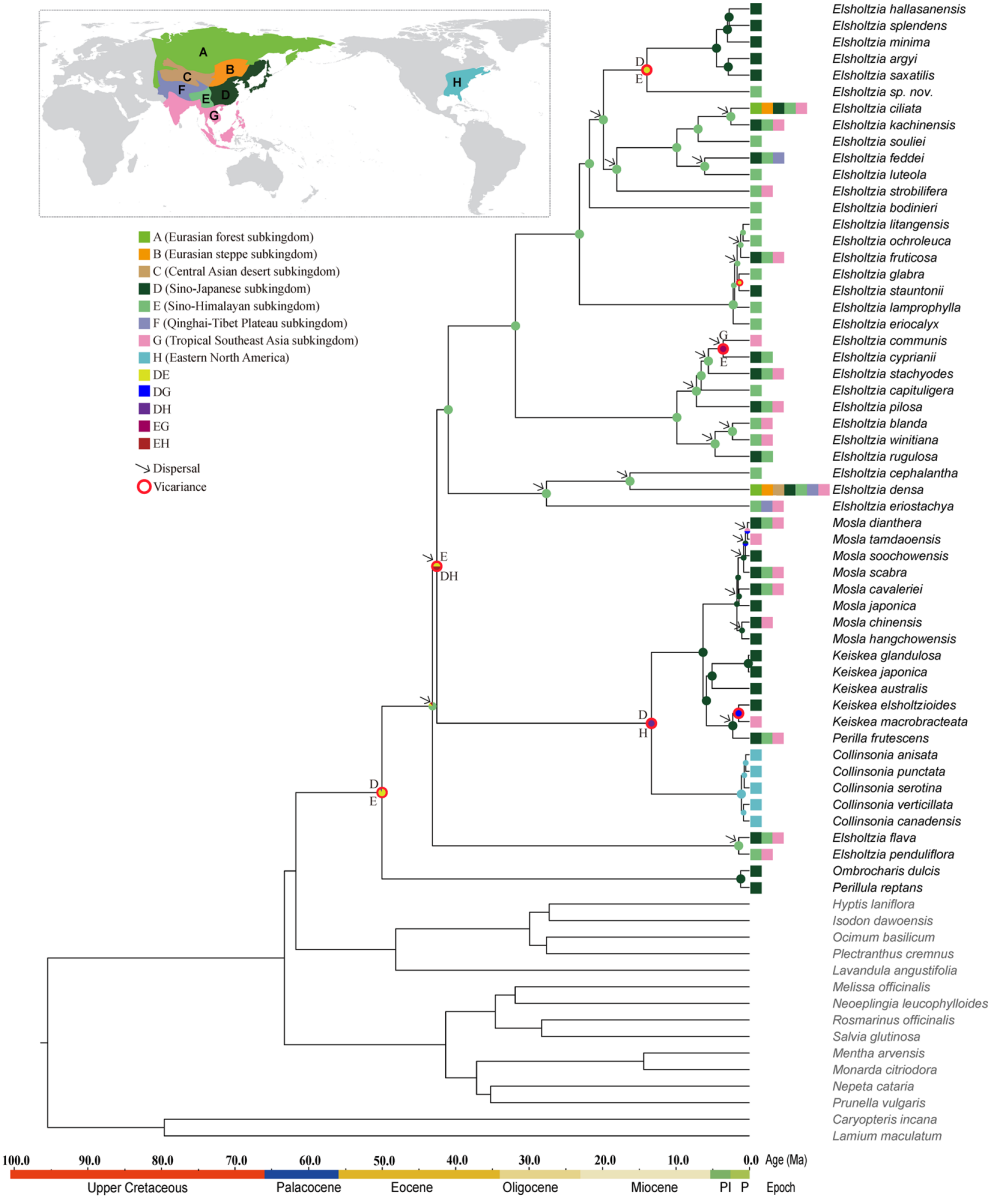
Ancestral area reconstruction of Elsholtzieae as inferred with Statistical Dispersal-Vicariance Analysis (S-DIVA) in rasp, using 1,000 random Bayesian trees from the beast analysis. Nodal probabilities of ancestral areas are depicted on the Bayesian tree of Fig. 2. Single-colored pie diagrams indicate an ancestor confined to a single geographic area; multi-colored pie diagrams represent the probabilities of different areas at each node. Arrows and circles represent dispersal and vicariance events, respectively. The base map was freely downloaded from Wikimedia Commons online (https://commons.wikimedia.org/wiki/File:BlankMap-Equirectangular.svg), with subsequent modifications.

Results from lagrange and S-DIVA (using different maximum area numbers) were similar, and so we only present the S-DIVA result based on a maximum area number of 2 (Fig. 3). AAR analyses indicated the Sino-Himalayan subkingdom (E) and Sino-Japanese subkingdom (D) as the ancestral areas of the tribe Elsholtzieae, but identified the Sino-Himalayan subkingdom (E) as the most likely ancestral range of all the members except *Perillula* and *Ombrocharis*. There were two important dispersals (from E to D then to H, or from E to H then to D), subsequently, which led to the colonization of Elsholtzieae in the Sino-Japanese subkingdom (D) and Eastern North America (H); then the vicariance between zone D and zone H resulted in the divergence between the *Mosla*-*Keiskea*-*Perilla* clade and *Collinsonia*. AAR analyses unambiguously show that the Sino-Himalayan subkingdom (E), Sino-Japanese subkingdom (D) and Eastern North America (H) are the most likely ancestral ranges of core *Elsholtzia*, the *Mosla*-*Keiskea*-*Perilla* clade, and *Collinsonia*, respectively. The *Elsholtzia* species that occur in the Sino-Japanese subkingdom (D) or Tropical Southeast Asia subkingdom (G) all had an origin in the Sino-Himalayan subkingdom (E). S-DIVA analysis indicated 13 dispersals and three subsequent vicariance events.

## Discussion

### Phylogenetic relationships within the tribe Elsholtzieae and systematic implications

The Japanese monotypic genus *Perillula* has not received much systematic attention since its assignment to subfamily Nepetoideae and tribe Elsholtzieae^25^. In agreement with recent studies^13^, our phylogenetic results support a sister relationship of *Perillula reptans* with the enigmatic genus *Ombrocharis* (Clade V, Fig. 1). The two genera form a clade that is sister to all other taxa of Elsholtzieae, and our molecular dating analyses indicate that the separation between these two extant species occurred only recently, during the Early Pleistocene (1.2 Ma, see node 6 in Fig. 2). Such a close relationship could be used to support the opinion that *Perillula* and *Ombrocharis* are more appropriately treated as two species of one genus, rather than two monotypic genera. The combination of the two genera is also supported by both palynological and morphological characteristics^13^. For example, they have similar pollen and mericarp morphology^13^, and also bear many additional morphological similarities such as short, tuber-like rhizomes; 6-flowered verticillasters in terminal racemoid thyrses; a campanulate, 11-veined, 2-lipped, and 5-lobed (3/2) calyx; a campanulate, slightly bilabiate, and 5-lobed (2/3) corolla; longer and included anterior stamens; and ± parallel and separate thecae with a small connective^26–28^. In fact, the differences between them, other than disjunct geographic distribution, are rather trivial in comparison to other members of Elsholtzieae, and certainly not large enough to warrant generic distinction.

Our cpDNA and nrDNA phylogenies show that the eastern North American *Collinsonia* (Clade III, Fig. 1) is monophyletic and sister to an eastern Asian *Mosla*-*Keiskea*-*Perilla* clade (Clade II, Fig. 1). Within the eastern Asian *Mosla*-*Keiskea*-*Perilla* clade, the monophyly of *Mosla* is robustly supported (100%/100%/1, Fig. 1), in agreement with previous pollen morphological and taxonomic studies^29, 30^. *Keiskea* was previously merged into *Collinsonia*, citing the laciniate lower corolla lobe of *Collinsonia* sensu stricto as the only morphological distinction between the two genera^1, 15^, but our molecular phylogenetic analyses of both cpDNA and nrDNA data do not support the proposed merger of these taxa. Instead, our finding is congruent with former phylogenetic^9, 13, 31^, karyotypic (*K. japonica*: 2*n* = 20 versus *C. canadensis*: 2*n* = 50, 52)^32–34^ and palynological studies (*Keiskea*: well-developed bireticulate, often forming large lumina by supratectal ridges versus *Collinsonia*: without supratectal ridges, mostly perforate or a faint/very weak bi-reticulate appearance)^16, 30^ that emphasize the distinction of *Collinsonia* from *Keiskea*. However, we concede that the latter genus is not monophyletic in our resulting cladograms. Furthermore, the economically important monotypic genus *Perilla* is embedded within part of *Keiskea*, and strongly supported as sister to the pair *K. elsholtzioides* + *K. macrobracteata* (100%/100%/1). This result is unexpected because *Perilla frutescens* has been previously thought to be most closely related to *Mosla* based on palynological characteristics^30^. Although the topology within Clade II is not fully supported by our current data, it seems clear that *Keiskea* is not monophyletic, and in need of taxonomic revision.

As the largest and most diversified genus in Elsholtzieae, it is not entirely surprising to find that *Elsholtzia* is also not monophyletic. Our phylogenetic analyses divided the genus *Elsholtzia* into two strongly supported clades: (1) *E. flava* + *E. penduliflora* (Clade IV), and (2) all other *Elsholtzia* species (Clade I, which we have divided into four subclades, Fig. 1). The pollen morphology of 18 species (32 specimens) in the genus *Elsholtzia* was investigated and three easily distinguished pollen types were found: perforate (*E. densa* Benth.), rugulose-bireticulate (*E. flava* Benth.), and bireticulate (16 other studied species)^35^. These types correspond well with our three major groups: Clade IV, Clade I Subclade IV, and Clade I Subclades I–III. To date, chromosome numbers have been reported for only twelve species in *Elsholtzia*, which are mostly diploids with the same number of chromosomes [2*n* = 2x = 16 or infrequently 18]^36^. They are occasionally tetraploids [e.g., *E. ciliata* (Thunb.) Hylander and *E. densa* Benth. var. *ianthina* (Maxim. ex Kanitz) C. Y. Wu & S. C. Huang (=*E. densa*), 2*n* = 4x = 32]^36–38^. However, it is interesting to note that the chromosome number (2*n* = 20) of *E. flava* and *E. penduliflora* (Clade IV) is reportedly quite different from that of the remainder of *Elsholtzia* species^36, 39^. The pair of *E. flava* and *E. penduliflora* also exhibit unique morphological traits compared to other species in the genus (e.g., stamens attached to the upper lip, the digitation of flower disc shorter than the ovary, nutlets attached near the base)^12^. Based on our molecular phylogenetic results and the morphological and cytological characteristics discussed above, we would support the separation of *E. flava* and *E. penduliflora* from other species of *Elsholtzia* as an independent genus.

### Diversification history

The crown age of subfamily Nepetoideae was estimated to be 63.4 Ma (95% HPD: 53.8–74.4 Ma), which correlates closely with the Cretaceous–Paleogene (K–Pg) boundary (Fig. 2). Our estimate is older than a previously published estimation (57.6 Ma, 95% HPD: 52.4–63.7 Ma) with the same two calibration points^11^. This incongruence is most likely due to their incomplete taxon sampling within the tribe Elsholtzieae by previous authors (only one *Elsholtzia* and one *Collinsonia* species were sampled). Curiously, the origin and early differentiation of Nepetoideae (the largest subfamily of Lamiaceae) corresponds in age with studies of a few other plant lineages, including epiphytic ferns^40^ and Menispermaceae^41^. Our refined time estimates and ancestral area reconstruction are important because they suggest an East Asian origin of the tribe Elsholtzieae at around 50.1 Ma, followed by subsequent and rapid diversification into all the major clades between 43–41 Ma (Figs 2, 3). The uplift and formation of the Himalayas and the Tibetan Plateau began at approximately this same time, about 50 Ma^42^. In addition, the paleobotanical evidence indicates that in the Early to Middle Eocene (50–48 Ma) a significant cooling occurred, followed by two warm intervals (46–43 Ma and 37–34 Ma) separated by a cool interval (42–38 Ma) in the Middle to Late Eocene^43, 44^. The uplift and formation of the Himalayas and the Tibetan Plateau alongside a gradual cooling climate globally after 50 Ma in the Eocene^45^ may well explain the origin and early diversification of Elsholtzieae into its major extant clades. For *Elsholtzia* sensu stricto (Clade I, Fig. 1), the semi-logarithmic lineage-through-time (LTT) plot indicates an accelerated lineage accumulation after ca. 20 Ma (Fig. 2). Most of the *Elsholtzia* species (26/41) occur in the Sino-Himalayan subkingdom, so one contributing factor in this fast diversification phase has almost certainly been the uplift of the QTP, which is thought to have occurred ca. 25–17, 15–13, 8–7, and 3.6–1.8 Ma^42, 46–48^. Each of the uplifts is also considered to have triggered other unrelated plant radiations within the QTP (e.g., *Ligularia*-*Cremanthodium*-*Parasenecio* complex^49^; *Caragana* ^50^; *Cyananthus* ^51^; *Lilium* ^52^; *Tibetia* and *Gueldenstaedtia* ^53^). The reconstructed phylogenies based on nrDNA, cpDNA or a combination of both datasets, suggest that the woody *Elsholtzia* species occurring in the Himalayas and the Tibetan Plateau are nested within herbaceous ones (Fig. 1) and thus most likely evolved from herbaceous ancestors. Hence, the uplift of the QTP not only promoted species radiation of *Elsholtzia*, but also induced the secondary feature of woodiness in a few lineages (in subclade II & III, Fig. 1) in response to strong selection pressures, possibly similar to those acting on island floras^24^. However, the rate of diversification for the different unrelated northern temperate groups (*Collinsonia*, *Mosla*, *Keiskea*, etc.) reached a high point until at least the Late Miocene (Fig. 2). Clearly, this high diversification was most likely initiated by the transition from a globally warm to a cooler climate towards the end of the Miocene and Quaternary climatic fluctuations^45, 54, 55^.

### Intercontinental disjunction within Elsholtzieae

Elsholtzieae is certainly of East Asian origin, as indicated by ancestral area reconstruction (Fig. 3). In general, the wide biogeographic distribution of Elsholtzieae species across most of Asia must be interpreted as a result of migration across the landscape, via existing land bridges and/or the rarer, long-distance dispersal events across land or water barriers, because the dispersal capacity of their nutlets is limited^1, 56^. The inferred dispersal events have certainly contributed to the tribe’s current distribution across East Asia and tropical Southeast Asia, but there is evidence of vicariance as well (Fig. 3). The stem and crown age of the East Asian *Mosla*-*Keiskea*-*Perilla* clade and the eastern North American *Collinsonia* clade was dated from 13.3 Ma to 42.6 Ma (Fig. 2), with an ancestral area of both Asia and North America (DEH or DH in Fig. 3) implying range extension to North America through the Beringian Land Bridge (BLB) sometime after the Middle Eocene. Throughout these periods, the BLB was certainly available as a route of migration. Soon afterward, in the Middle Miocene (Fig. 3), a vicariance event triggered cladogenesis between the sister lineages of Old World *Mosla*-*Keiskea*-*Perilla* and New World *Collinsonia* from a common ancestor that inhabited both continents. Our estimate of divergence time of Old World *Mosla*-*Keiskea*-*Perilla* and New World *Collinsonia* is consistent with the palaeoclimate and fossil evidence. The Miocene was a period with globally warmer climates than those in the preceding Oligocene, or the subsequent Pliocene^2^. Extensive fossil evidence from the Early to Middle Miocene reveals that diverse, temperate, deciduous, and mesophytic vegetation was widely distributed in the Northern Hemisphere^57–61^. Later in the Middle Miocene (13 to 18 Ma) a distinct climatic cooling period may have resulted in the range reduction of a once more or less continuously distributed temperate forest across the Northern Hemisphere^62, 63^. The Miocene floras had many elements in common with the modern mesophytic floras of eastern Asia and eastern North America^64^, supporting the proposal that the divergence of the modern northern temperate elements occurred during that period. However, the glaciation events of the Pleistocene would have induced subsequent allopatic speciation on both continents. Consistent with this hypothesis, more recent diversification events in the *Mosla*-*Keiskea*-*Perilla*-*Collinsonia* clade were dated from the mid-Pliocene to mid-Pleistocene.

## Materials and Methods

### Taxon sampling

The ingroup included 70 accessions of 59 taxa from Elsholtzieae (Appendix 1 and 2). Our sampling contained all seven genera within Elsholtzieae^13^, and all three sections of *Elsholtzia* recognized by Press^65^. We also covered all three sections of *Elsholtzia*, both of the two subsections, all of the eight series in a different classification system proposed by Wu & Huang^66^. To place the tribe Elsholtzieae within a broader phylogenetic framework of the subfamily Nepetoideae (Lamiaceae), and to take advantage of molecular clock calibration points used in previous studies^10, 11^, we also downloaded DNA sequences of eight Mentheae and five Ocimeae species from GenBank. Based on previous molecular phylogenetic studies of the family^4, 10^, *Caryopteris incana* (Thunb.) Miq. and *Lamium maculatum* L. were selected as outgroups since they are members of two different subfamilies placed within a common clade that is sister to Nepetoideae^4^. In total, therefore, 85 accessions were included in this study (Appendix 1). For both cp and nr DNA data sets, we were able to amplify and sequence most of the target loci from all of the samples, although a few taxa have missing data from one to four fragments due to the low quality of DNA extracted from herbarium samples or lack of specific primers.

### Laboratory work and data handling

Total genomic DNA was extracted from either silica-dried plant material or herbarium specimens following the modified CTAB method^67^. The gene amplification by PCR procedures were similar to those described in a previous study^11^. PCR products were obtained with JumpStart^TM^ Taq ReadyMix^TM^ (Saint Louis, Missouri, USA). The plastid genes *rbcL* and *matK* were amplified and sequenced following the protocol provided by CBOL (http://www.barcoding.si.edu/pdf/informationonbarcodeloci.pdf). PCR amplification and sequencing for *trnL-F* used the ‘C’ and ‘F’ primers^68^. The *ycf1* region has proved its great phylogenetic utility within Lamiaceae^10^, but in our study the ‘ycf4887f’ and ‘ycf5778r’ primers were excluded because of unsuccessful amplification in most samples. The nuclear ribosomal internal transcribed spacer (ITS) was amplified and sequenced using the primers ‘Leu1’^69^ and ‘ITS4’^70^. The nuclear ribosomal external transcribed spacer (ETS) was amplified and sequenced using the primers ‘18S-IGS’^71^ and ‘ETS-bdf1’^10^. Sequences were generated with an ABI 377XL DNA sequencer, and then edited, assembled, and aligned using Geneious v4.8.5^72^. New sequences were submitted to GenBank (Appendix 1). The pairwise distances among species were estimated using the software mega version 7^73^.

### Phylogenetic analyses

Maximum parsimony (MP), maximum likelihood (ML) and Bayesian inference (BI) analyses were performed for the cpDNA (five regions) and nrDNA (two loci) sequence data separately (*rbcL*: 501 bp, *matK*: 885 bp, *trnL-F*: 934 bp, *ycf1*: 4275 bp, *ycf1-rps15*: 733 bp, ITS: 695 bp and ETS: 441 bp). Although the result of the incongruence length difference (ILD) test^74^ revealed significant incongruences between cp and nr datasets (*P*  =  0.001), it is important to remember that the ILD is meant to be an overall measure of incongruence between datasets. The global nature of the test may contribute to at least two hypothetical misinterpretations or errors^75^. A measure of local incongruence that inspects the tree on a node-by-node basis might be able to address the analytical limitations of the global test^75^. A comparison of the individual topologies showed that there were no supported topological incongruencies among datasets [cp, nr (ETS, ITS), combined] (visually assessed: >70% MP bootstrap percentage, MPBP; >85% ML bootstrap percentage, MLBP; >0.9 Bayesian posterior probability, PP). For this reason, the two datasets were concatenated into a single matrix.

Then MP analyses were conducted on the concatenated dataset using paup* v.4.0b10^76^ by sampling 1,000 random addition replicates with TBR branch swapping and Multrees on. Bootstrap^77^ values were obtained by performing 1,000 heuristic searches using all characters, with 10 TBR branch swapping replicates per bootstrap, and saving no more than 100 trees per replicate. ML and BI analyses were also conducted on the concatenated dataset, but with partitions (7: *ycf1*, *matK*, *rbcL*, *trnL-F*, *ycf1-rps15*, ETS and ITS; 3: cp, ETS and ITS; 2: cp and nr). However, there were no topological differences discovered among the results based on these three partitioning strategies.

ML analyses were implemented with raxml-HPC2 on XSEDE^78^ on the CIPRES Science Gateway v.3.3 (http://www.phylo.org/)^79^. A partitioned model was selected, and 1,000 bootstrap iterations were conducted, with other parameters using the default settings.

Partitioned BI analyses were run with mrbayes v.3.2.6^80^ implemented in the CIPRES platform. Models for each partition were selected based on the AIC criterion as implemented in jmodeltest 2.1.3^81^. The best-fitting models of sequence evolution were TPM2uf + I + G (*rbcL*), GTR + I + G (ITS, cp, nr), TVM + G (*ycf1*, *matK*) and GTR + G (*trnL-F*, *ycf1-rps15* and ETS). For those models (TPM2uf + I + G, TVM + G) that are not implemented in mrbayes, we followed the software manual’s recommendation to substitute the preferred model with the next more complex model available in mrbayes (which is HKY + I + G and GTR + G, repectively). Two independent parallel runs of four Metropolis-coupled Monte Carlo Markov Chains (MCMCs) were run, sampling every 2000th generations for 20 million total generations. The convergence of the parameters was assessed with tracer v.1.6.0^82^. Effective sample sizes (ESS), potential scale reduction factor (PSRF) values, and average standard deviations of split frequencies (asdsf) were well within acceptable ranges (ESS ≫ 200, PSRF between 1.000 and 1.001, asdsf <0.01). After discarding the first 10% of the sampled trees as burn-in, a majority rule consensus tree and posterior probabilities (PP) of bipartitions were computed using the remaining trees.

### Divergence time estimation

Although Lamiaceae are not well represented in the fossil record^1^, we can confidently use the following two fossils as calibration points. The first calibration point is based on the hexacolpate and three-nucleate pollen fossil from Early Eocene sediments in India, which was identified as *Ocimum* ^83^. As hexacolpate and three-nucleate pollen was considered to be a synapomorphy for subfamily Nepetoideae^1^, the fossil was suggested to place at the crown of Nepetoideae (node1 in Fig. 2) as opposed to elsewhere (crown of the Ocimeae)^11^. For this calibration point, we used a lognormal distribution, with an offset at 49 Ma, a mean of 2.6 Ma, and a standard deviation (SD) of 0.5 Ma^11^. The assigned mean of 2.6 Ma to the offset of 49 Ma allowed for the possibility that the Coniacian hexacolpate fossil described by Boltenhagen (pending taxa)^84, 85^ is truly Nepetoideae. The second calibration point is based on a fruit fossil of *Melissa* from the Early-Middle Oligocene^86, 87^, which was assigned to constrain the most recent common ancestor (MRCA) of *Melissa* and *Neoeplingia* with a lognormal distribution (node2 in Fig. 2: offset, 28.4 Ma; mean, 1.5 Ma; SD, 0.5 Ma)^11^.

Divergence time analyses were performed using beast v.2.3.2^88^ implemented in the CIPRES portal. A Yule model and the relaxed lognormal clock model^89^ were selected, and GTR + G substitution model was set for each partition, except for ITS (GTR + I + G) and *rbcL* (HKY + I + G). Parameters were estimated using two independent runs of 100 million generations, with sampling every 10,000 generations. We initially explored dating with two different datasets: (1) simplified concatenated dataset (cp + nr, 7 partitions; 69 accessions, without redundant ones; small gaps and difficult-to-align areas removed) and (2) simplified cp dataset (5 partitions; 69 accessions; small gaps and difficult-to-align areas removed). Among these analyses, most divergence times did not vary more than c. 5% across the resulting chronograms. Thus we will only present the results base on the simplified concatenated dataset (#1 above). Once this dataset had been selected, we then initiated three more independent beast analyses with it to check for repeatability and consistency of results. In each run, all tree and coalescent model parameters converged, judged by ESS values (>200) after setting the burn-in to 10%. tracer v.1.6.0 was used to examine the sampling adequacy and convergence of the chains to a stationary distribution. We combined the tree files of the four runs (burn-in 20% each) in logcombiner v.2.4.4, and used treeannotator v.2.4.4 to summarize the post burn-in trees and produce a maximum clade credibility chronogram showing mean divergence time estimates with 95% HPD intervals^88^, which was visualized in figtree v.1.4.2. To visualize the temporal dynamics of diversification of Elsholtzieae, we generated standard LTT plots in ape v.3.4^90^.

### Ancestral area reconstructions

Ancestral area reconstructions (AAR) and estimation of spatial patterns of geographic diversification within tribe Elsholtzieae were done with Statistical Dispersal-Vicariance Analysis (S-DIVA)^91, 92^ and lagrange ^93^ as implemented in rasp v.3.2^94^. Biogeographic data were compiled from distributions documented in the systematic literature and based on herbarium records. Species distributed in Asia were assigned to one or more of seven zones/subkingdoms according to the floristic divisions of East Asia^95^. These areas comprise: (A) Eurasian forest subkingdom, (B) Eurasian steppe subkingdom, (C) Central Asian desert subkingdom, (D) Sino-Japanese subkingdom, (E) Sino-Himalayan subkingdom, (F) Qinghai-Tibet Plateau subkingdom and (G) Tropical Southeast Asia subkingdom (Fig. 3). We coded Eastern North America (H) as a separate zone. Subsets of 1,000 random Bayesian trees from the beast analysis were used to estimate probabilities of ancestral areas at each node. We removed the taxa classified in tribe Ocimeae (5), Mentheae (5) and the other outgroups (2) from the AAR analyses due to the fact that we included only placeholder species for those lineages, and therefore the difficulty in coding them within appropriate ranges. In S-DIVA, we used the maxareas option (all possible, 4, and 2 areas) to explore the impact of restricting the number of unit areas allowed in ancestral distributions. AARs for all nodes were visualized on each of the single Bayesian trees obtained in the Bayesian runs. In lagrange, dispersal probabilities range from 0.5 for separated areas (East Asia vs. Eastern North America) to 0.75 for connected areas (through the Beringian land bridge) to 1.0 for contiguous areas, and are provided for three time intervals (0–5 Ma, 5–30 Ma, and 30–50.1 Ma) spanning the estimated time frames for evolution of tribe Elsholtzieae. All ancestral ranges of two combined areas were permitted based on extant and potentially plausible ranges.

## Electronic supplementary material

Supplementary Information
