# Supplementary material for: Molecular phylogenetics and biogeography of the mint tribe Elsholtzieae (Nepetoideae, Lamiaceae), with an emphasis on its diversification in East Asia
Source: Sci Rep. 2017 May 17;7:2057. doi: 10.1038/s41598-017-02157-6 (PMC5435694; doi:10.1038/s41598-017-02157-6)
Supplement: Supplementary file 1 — Supplementary Information [file 41598_2017_2157_MOESM1_ESM.pdf]

# Molecular phylogenetics and biogeography of the mint tribe Elsholtzieae (Nepetoideae, Lamiaceae), with an emphasis on its diversification in East Asia

Pan Li[1,#], Zhe-Chen Qi[2,#], Lu-Xian Liu[1], Tetsuo Ohi-Toma[3], Joongku Lee[4], Tsung-Hsin Hsieh[5], Cheng-Xin Fu[1], Kenneth M. Cameron[6], Ying-Xiong Qiu[1,*]

[1]Key Laboratory of Conservation Biology for Endangered Wildlife of the Ministry of Education, and Laboratory of Systematic & Evolutionary Botany and Biodiversity, College of Life Sciences, Zhejiang University, Hangzhou 310058, People's Republic of China. [2]College of Life Sciences, Zhejiang Sci-Tech University, Hangzhou 310018, People's Republic of China. [3]Botanical Gardens, Graduate School of Science, The University of Tokyo, Tokyo 112-0001, Japan. [4]Department of Environment and Forest Resources, Chungnam National University, Daejeon 34134, South Korea. [5]Department of Ecoscience and Ecotechnology, National University of Tainan, Tainan 700, Republic of China. [6]Department of Botany, University of Wisconsin, Madison, Wisconsin 53706, USA. [#]These authors contributed equally to this work. [*]Correspondence and requests for materials should be addressed to Y.X.Q. (qyxhero@zju.edu.cn)

Appendix 1 List of species included in this study with their classification, authorities and GenBank accession numbers for all sequences.
Sequences newly obtained are indicated by an asterisk (*), missing sequences are indicated by a double slash (//).

| Taxon | ITS | ETS | ycf1 | ycf1-rps15 | trnL-F | rbcL | matK |
|---|---|---|---|---|---|---|---|
| **Elsholtzieae** | | | | | | | |
| *Collinsonia anisata* Sims | *KY552484 | *KY552552 | *KY625114 | // | *KY624984 | *KY624915 | // |
| *Collinsonia canadensis* L. | *KY552485 | *KY552553 | *KY625115 | *KY625052 | *KY624985 | *KY624916 | *KY624850 |
| *Collinsonia canadensis* L. | *KY552486 | *KY552554 | *KY625116 | *KY625053 | *KY624986 | *KY624917 | *KY624851 |
| *Collinsonia canadensis* L. | *KY552487 | *KY552555 | *KY625117 | *KY625054 | *KY624987 | *KY624918 | *KY624852 |
| *Collinsonia punctata* Elliott | *KY552488 | *KY552556 | *KY625118 | // | // | *KY624919 | // |
| *Collinsonia serotina* Walter | *KY552489 | *KY552557 | *KY625119 | *KY625055 | *KY624988 | *KY624920 | *KY624853 |
| *Collinsonia verticillata* Baldwin ex Elliott | *KY552490 | *KY552558 | *KY625120 | *KY625056 | *KY624989 | *KY624921 | *KY624854 |
| *Elsholtzia argyi* H.Lév. | *KY552491 | *KY552559 | *KY625121 | *KY625057 | *KY624990 | *KY624922 | *KY624855 |
| *Elsholtzia blanda* (Benth.) Benth. | *KY552492 | *KY552560 | *KY625122 | *KY625058 | *KY624991 | *KY624923 | *KY624856 |
| *Elsholtzia bodinieri* Vaniot | *KY552493 | *KY552561 | *KY625123 | *KY625059 | *KY624992 | *KY624924 | *KY624857 |
| *Elsholtzia capituligera* C.Y.Wu | *KY552494 | *KY552562 | *KY625124 | *KY625060 | *KY624993 | *KY624925 | *KY624858 |
| *Elsholtzia cephalantha* Hand.-Mazz. | *KY552495 | *KY552563 | *KY625125 | *KY625061 | *KY624994 | *KY624926 | *KY624859 |
| *Elsholtzia ciliata* (Thunb.) Hyl. | *KY552496 | *KY552564 | *KY625126 | *KY625062 | *KY624995 | *KY624927 | *KY624860 |
| *Elsholtzia communis* (Collett & Hemsl.) Diels | *KY552497 | *KY552565 | *KY625127 | *KY625063 | *KY624996 | *KY624928 | *KY624861 |
| *Elsholtzia cyprianii* (Pavol.) C.Y.Wu & S.Chow | *KY552498 | *KY552566 | *KY625128 | *KY625064 | *KY624997 | *KY624929 | *KY624862 |
| *Elsholtzia cyprianii* var. *angustifolia* C.Y.Wu & S.C.Huang | *KY552499 | *KY552567 | *KY625129 | *KY625065 | *KY624998 | *KY624930 | *KY624863 |
| *Elsholtzia densa* Benth. | *KY552500 | *KY552568 | *KY625130 | *KY625066 | *KY624999 | *KY624931 | *KY624864 |
| *Elsholtzia densa* Benth. | *KY552501 | *KY552569 | *KY625131 | *KY625067 | *KY625000 | *KY624932 | *KY624865 |
| *Elsholtzia densa* var. *ianthina* (Maxim. ex Kanitz) C.Y.Wu & S.C.Huang | *KY552502 | *KY552570 | *KY625132 | *KY625068 | *KY625001 | *KY624933 | *KY624866 |
| *Elsholtzia densa* var. *ianthina* (Maxim. ex Kanitz) | *KY552503 | *KY552571 | *KY625133 | *KY625069 | *KY625002 | *KY624934 | *KY624867 |

| | | | | | | | | |
|---|---|---|---|---|---|---|---|---|
| | C.Y.Wu & S.C.Huang | | | | | | | |
| | *Elsholtzia eriocalyx* C.Y.Wu & S.C.Huang | *KY552504 | *KY552572 | *KY625134 | *KY625070 | *KY625003 | *KY624935 | *KY624868 |
| | *Elsholtzia eriostachya* (Benth.) Benth. | *KY552505 | *KY552573 | *KY625135 | *KY625071 | *KY625004 | *KY624936 | *KY624869 |
| | *Elsholtzia eriostachya* (Benth.) Benth. | *KY552506 | *KY552574 | *KY625136 | *KY625072 | *KY625005 | *KY624937 | *KY624870 |
| | *Elsholtzia feddei* H.Lév. | *KY552507 | *KY552575 | *KY625137 | *KY625073 | *KY625006 | *KY624938 | *KY624871 |
| | *Elsholtzia feddei* f. *robusta* C.Y.Wu & S.C.Huang | *KY552508 | *KY552576 | *KY625138 | *KY625074 | *KY625007 | *KY624939 | *KY624872 |
| | *Elsholtzia flava* Benth. | *KY552509 | *KY552577 | *KY625139 | *KY625075 | *KY625008 | *KY624940 | *KY624873 |
| | *Elsholtzia flava* Benth. | *KY552510 | *KY552578 | *KY625140 | *KY625076 | *KY625009 | *KY624941 | *KY624874 |
| | *Elsholtzia fruticosa* (D.Don) Rehder | *KY552511 | *KY552579 | *KY625141 | *KY625077 | *KY625010 | *KY624942 | *KY624875 |
| | *Elsholtzia glabra* C.Y.Wu & S.C.Huang | *KY552512 | *KY552580 | *KY625142 | *KY625078 | *KY625011 | *KY624943 | *KY624876 |
| | *Elsholtzia hallasanensis* Y.N.Lee | *KY552513 | *KY552581 | *KY625143 | *KY625079 | *KY625012 | *KY624944 | *KY624877 |
| | *Elsholtzia kachinensis* Prain | *KY552514 | *KY552582 | *KY625144 | *KY625080 | *KY625013 | *KY624945 | *KY624878 |
| | *Elsholtzia lamprophylla* C. L.Xiang & E.D.Liu | *KY552515 | // | *KY625145 | *KY625081 | *KY625014 | *KY624946 | *KY624879 |
| | *Elsholtzia litangensis* C.X.Pu & W.Y.Chen | *KY552516 | *KY552583 | *KY625146 | *KY625082 | *KY625015 | *KY624947 | *KY624880 |
| | *Elsholtzia luteola* Diels | *KY552517 | *KY552584 | *KY625147 | *KY625083 | *KY625016 | *KY624948 | *KY624881 |
| | *Elsholtzia minima* Nakai | *KY552518 | *KY552585 | *KY625148 | *KY625084 | *KY625017 | *KY624949 | *KY624882 |
| | *Elsholtzia ochroleuca* Dunn | *KY552519 | *KY552586 | *KY625149 | *KY625085 | *KY625018 | *KY624950 | *KY624883 |
| | *Elsholtzia ochroleuca* var. *parvifolia* C.Y.Wu & S.C.Huang | *KY552520 | *KY552587 | *KY625150 | *KY625086 | *KY625019 | *KY624951 | *KY624884 |
| | *Elsholtzia penduliflora* W.W.Sm. | *KY552521 | *KY552588 | *KY625151 | *KY625087 | *KY625020 | *KY624952 | *KY624885 |
| | *Elsholtzia penduliflora* W.W.Sm. | *KY552522 | *KY552589 | *KY625152 | *KY625088 | *KY625021 | *KY624953 | *KY624886 |
| | *Elsholtzia pilosa* (Benth.) Benth. | *KY552523 | *KY552590 | *KY625153 | *KY625089 | *KY625022 | *KY624954 | *KY624887 |
| | *Elsholtzia rugulosa* Hemsl. | *KY552524 | *KY552591 | *KY625154 | *KY625090 | *KY625023 | *KY624955 | *KY624888 |
| | *Elsholtzia saxatilis* (Kom.) Nakai ex Kitag. | *KY552525 | *KY552592 | *KY625155 | *KY625091 | *KY625024 | *KY624956 | *KY624889 |
| | *Elsholtzia souliei* H.Lév. | *KY552526 | *KY552593 | *KY625156 | // | *KY625025 | *KY624957 | // |
| | *Elsholtzia splendens* Nakai ex F.Maek. | *KY552527 | *KY552594 | *KY625157 | *KY625092 | *KY625026 | *KY624958 | *KY624890 |

| | | | | | | | |
|---|---|---|---|---|---|---|---|
| *Elsholtzia splendens* Nakai ex F.Maek. | *KY552528 | *KY552595 | *KY625158 | *KY625093 | *KY625027 | *KY624959 | *KY624891 |
| *Elsholtzia stachyodes* (Link) Raizada & H.O.Saxena | *KY552529 | *KY552596 | *KY625159 | *KY625094 | *KY625028 | *KY624960 | *KY624892 |
| *Elsholtzia stauntonii* Benth. | *KY552530 | *KY552597 | *KY625160 | *KY625095 | *KY625029 | *KY624961 | *KY624893 |
| *Elsholtzia strobilifera* (Benth.) Benth. | *KY552531 | *KY552598 | *KY625161 | *KY625096 | *KY625030 | *KY624962 | *KY624894 |
| *Elsholtzia winitiana* Craib | *KY552532 | *KY552599 | *KY625162 | *KY625097 | *KY625031 | *KY624963 | *KY624895 |
| *Elsholtzia* sp. nov. | *KY552533 | *KY552600 | *KY625163 | *KY625098 | *KY625032 | *KY624964 | *KY624896 |
| *Keiskea australis* C.Y.Wu & H.W.Li | *KY552534 | *KY552601 | *KY625164 | *KY625099 | *KY625033 | *KY624965 | *KY624897 |
| *Keiskea elsholtzioides* Merr. | *KY552535 | *KY552602 | *KY625165 | *KY625100 | *KY625034 | *KY624966 | *KY624898 |
| *Keiskea elsholtzioides* Merr. | *KY552536 | *KY552603 | *KY625166 | *KY625101 | *KY625035 | *KY624967 | *KY624899 |
| *Keiskea glandulosa* C.Y.Wu | // | *KY552604 | *KY625167 | // | *KY625036 | *KY624968 | *KY624900 |
| *Keiskea japonica* Miq. | *KY552537 | *KY552605 | *KY625168 | *KY625102 | *KY625037 | *KY624969 | *KY624901 |
| *Keiskea japonica* Miq. | *KY552538 | *KY552606 | *KY625169 | *KY625103 | *KY625038 | *KY624970 | *KY624902 |
| *Keiskea macrobracteata* Masam. | *KY552539 | *KY552607 | *KY625170 | // | *KY625039 | *KY624971 | // |
| *Mosla cavaleriei* H.Lév. | *KY552545 | *KY552608 | *KY625171 | *KY625104 | *KY625040 | *KY624972 | *KY624903 |
| *Mosla chinensis* Maxim. | *KY552540 | *KY552609 | *KY625172 | *KY625105 | *KY625041 | *KY624973 | *KY624904 |
| *Mosla dianthera* (Buch.-Ham. ex Roxb.) Maxim. | *KY552544 | *KY552610 | *KY625173 | *KY625106 | *KY625042 | *KY624974 | *KY624905 |
| *Mosla hangchowensis* Matsuda | *KY552541 | *KY552611 | *KY625174 | *KY625107 | *KY625043 | *KY624975 | *KY624906 |
| *Mosla japonica* (Benth. ex Oliv.) Maxim. | *KY552546 | *KY552612 | *KY625175 | // | *KY625044 | *KY624976 | *KY624907 |
| *Mosla scabra* (Thunb.) C.Y.Wu & H.W.Li | *KY552542 | *KY552613 | *KY625176 | *KY625108 | *KY625045 | *KY624977 | *KY624908 |
| *Mosla soochowensis* Matsuda | *KY552547 | *KY552614 | *KY625177 | *KY625109 | *KY625046 | *KY624978 | *KY624909 |
| *Mosla tamdaoensis* Phuong | *KY552543 | *KY552615 | *KY625178 | // | *KY625047 | *KY624979 | *KY624910 |
| *Ombrocharis dulcis* Hand.-Mazz. | KT210250 | KT210223 | KT210358 | KT210307 | KT210334 | KT210258 | // |
| *Perilla frutescens* (L.) Britton | *KY552548 | *KY552616 | *KY625179 | *KY625110 | *KY625048 | *KY624980 | *KY624911 |
| *Perilla frutescens* var. *auriculato-dentata* C.Y.Wu & S.J.Hsuan ex H.W.Li | *KY552549 | *KY552617 | *KY625180 | *KY625111 | *KY625049 | *KY624981 | *KY624912 |
| *Perillula reptans* Maxim. | *KY552550 | *KY552618 | *KY625181 | *KY625112 | *KY625050 | *KY624982 | *KY624913 |

| | | | | | | | | |
|---|---|---|---|---|---|---|---|---|
| | *Perillula reptans* Maxim. | *KY552551 | *KY552619 | *KY625182 | *KY625113 | *KY625051 | *KY624983 | *KY624914 |
| **Ocimeae** | | | | | | | | |
| | *Hyptis laniflora* Benth. | JF301548 | JF304259 | JF289024 | JF289024 | JF301370 | // | // |
| | *Isodon dawoensis* (Hand.-Mazz.) H. Hara | KF855429 | // | JF289025 | JF289025 | JF301372 | // | // |
| | *Lavandula angustifolia* Mill. | FJ593399 | // | JF289028 | JF289028 | AY570457 | Z37407 | HE967430 |
| | *Ocimum basilicum* L. | DQ667240 | // | JF289049 | JF289049 | AY570462 | KX015763 | KX096054 |
| | *Plectranthus cremnus* B.J. | // | // | JF289052 | JF289052 | JF301393 | // | // |
| **Mentheae** | | | | | | | | |
| | *Melissa officinalis* L. | JF301353 | JF301325 | JF289042 | JF289042 | JF301386 | KM360879 | KP172051 |
| | *Mentha arvensis* L. | JQ669115 | JQ669190 | JF289043 | JF289043 | JF301387 | KC571787 | KP172052 |
| | *Monarda citriodora* Cerv. ex Lag. | JQ669124 | JQ669200 | JF289045 | JF289045 | JF301388 | JX254905 | // |
| | *Neoeplingia leucophylloides* Ramamoorthy, Hiriart & Medrano | JF301354 | JF301327 | JF289047 | JF289047 | JF301390 | // | // |
| | *Nepeta cataria* L. | JQ669126 | JQ669202 | JF289048 | JF289048 | JF301391 | KT178127 | KT176606 |
| | *Prunella vulgaris* L. | JQ669130 | JQ669206 | JF289055 | JF289055 | DQ667508 | KP644119 | KJ593074 |
| | *Rosmarinus officinalis* L. | DQ667241 | JF301329 | JF289058 | JF289058 | AY570465 | KM360960 | KP172065 |
| | *Salvia glutinosa* L. | KJ584253 | KF307496 | JF289061 | JF289061 | AY570480 | Z37440 | KP852741 |
| **Outgroup** | | | | | | | | |
| | *Caryopteris incana* (Thunb. ex Houtt.) Miq. | EF508064 | *KY552620 | JF289003 | JF289003 | JF301359 | U28869 | AF315295 |
| | *Lamium maculatum* L. | KF055056 | // | JF289027 | JF289027 | JF301374 | Z37402 | AF531780 |

Appendix 2. Voucher information for species newly sequenced for this study, including collector's name and collection number, collection locality and country, and herbarium acronym where the specimen is deposited.

| **Taxon** | ***Collector, Coll. N°** | **locality** | **Country** | **Herbarium** |
|---|---|---|---|---|
| *Caryopteris incana* (Thunb.) Miq. | Pan Li, PNLI20120421-1 | Yongkang, Zhejiang | China | HZU |
| *Collinsonia anisata* Sims | Steve L. Orzell and Edwin L. Bridges 16469 | Gadsden County, Florida | United States | FLAS |
| *Collinsonia canadensis* L. | Lucas C. Majure 2722 | Alachua County, Florida | United States | FLAS |
| *Collinsonia canadensis* L. | Pan Li, LP1007189 | Gill State Forest, North Carolina | United States | HZU |
| *Collinsonia canadensis* L. | Pan Li, LP150411-1 | Giles County, Virginia | United States | HZU |
| *Collinsonia punctata* Elliott | R. Kral 3705 | Leon County, Florida | United States | FLAS |
| *Collinsonia serotina* Walter | J. Richard Abbott 14105 | Suwannee County, Florida | United States | FLAS |
| *Collinsonia verticillata* Baldwin ex Elliott | Wilbur H. Duncan 18484 | Forsyth County, Georgia | United States | FLAS |
| *Elsholtzia argyi* H.Lév. | Pan Li, PNLI20120255 | Lin'an, Zhejiang | China | HZU |
| *Elsholtzia blanda* (Benth.) Benth. | Pan Li, PNLI20120028-1 | Yongde, Yunnan | China | HZU |
| *Elsholtzia bodinieri* Vaniot | Pan Li, PNLI20120404-1 | Fumin, Yunnan | China | HZU |
| *Elsholtzia capituligera* C.Y.Wu | Pan Li, PNLI20120291-1 | Shangri-La, Yunnan | China | HZU |
| *Elsholtzia cephalantha* Hand.-Mazz. | Pan Li, LP150610-1 | Kangle, Gansu | China | HZU |
| *Elsholtzia ciliata* (Thunb.) Hyl. | Pan Li, PNLI20120089 | Yichun, Heilongjiang | China | HZU |
| *Elsholtzia communis* (Collett & Hemsl.) Diels | Pan Li, PNLI20130568-1 | Firmenich Aromatics, Shanghai | China | HZU |
| *Elsholtzia cyprianii* (Pavol.) C.Y.Wu & S.Chow | Pan Li, PNLI20120304 | Yulong, Yunnan | China | HZU |
| *Elsholtzia cyprianii* var. *angustifolia* C.Y.Wu & S.C.Huang | Pan Li, PNLI20120303-1 | Yulong, Yunnan | China | HZU |
| *Elsholtzia densa* Benth. | Yongming Yuan, YGY2012-034① | Yuzhong, Gansu | China | HZU |
| *Elsholtzia densa* Benth. | Yongming Yuan, YGY2012-033① | Yuzhong, Gansu | China | HZU |
| *Elsholtzia densa* var. *ianthina* (Maxim. ex Kanitz) C.Y.Wu & S.C.Huang | Pan Li, LP150583-1 | Yuzhong, Gansu | China | HZU |
| *Elsholtzia densa* var. *ianthina* (Maxim. ex Kanitz) | Pan Li, LP150583-2 | Yuzhong, Gansu | China | HZU |

| | | | | |
|---|---|---|---|---|
| C.Y.Wu & S.C.Huang | | | | |
| *Elsholtzia eriocalyx* C.Y.Wu & S.C.Huang | Pan Li, PNLI20120300 | Yulong, Yunnan | China | HZU |
| *Elsholtzia eriostachya* (Benth.) Benth. | Pan Li, PNLI20120227-1 | Xiangcheng, Sichuan | China | HZU |
| *Elsholtzia eriostachya* (Benth.) Benth. | Pan Li, PNLI20120227-2 | Xiangcheng, Sichuan | China | HZU |
| *Elsholtzia feddei* H.Lév. | Pan Li, PNLI20120190 | Yajiang, Sichuan | China | HZU |
| *Elsholtzia feddei* f. *robusta* C.Y.Wu & S.C.Huang | Pan Li, PNLI20120192 | Yajiang, Sichuan | China | HZU |
| *Elsholtzia flava* Benth. | Pan Li, PNLI20120353 | Yanyuan, Sichuan | China | HZU |
| *Elsholtzia flava* Benth. | Pan Li, PNLI20120017-1 | Yongde, Yunnan | China | HZU |
| *Elsholtzia fruticosa* (D.Don) Rehder | Pan Li, PNLI20120267 | Eryuan, Yunnan | China | HZU |
| *Elsholtzia glabra* C.Y.Wu & S.C.Huang | Pan Li, PNLI20120332-1 | Yongsheng, Yunnan | China | HZU |
| *Elsholtzia hallasanensis* Y.N.Lee | Joongku Lee, PNLI20120474 | Jeju-do | South Korea | HZU |
| *Elsholtzia kachinensis* Prain | Pan Li, PNLI20120266 | Eryuan, Yunnan | China | HZU |
| *Elsholtzia lamprophylla* C. L.Xiang & E.D.Liu | Pan Li, LP150703-1 | Xiangcheng, Sichuan | China | HZU |
| *Elsholtzia litangensis* C.X.Pu & W.Y.Chen | Pan Li, PNLI20120220-1 | Litang, Sichuan | China | HZU |
| *Elsholtzia luteola* Diels | Pan Li, PNLI20120327-1 | Yulong, Yunnan | China | HZU |
| *Elsholtzia minima* Nakai | Joongku Lee, PNLI20120473 | Jeju-do | South Korea | HZU |
| *Elsholtzia ochroleuca* Dunn | Pan Li, PNLI20120401 | Fumin, Yunnan | China | HZU |
| *Elsholtzia ochroleuca* var. *parvifolia* C.Y.Wu & S.C.Huang | Pan Li, PNLI20120153 | Maerkang, Sichuan | China | HZU |
| *Elsholtzia penduliflora* W.W.Sm. | Pan Li, PNLI20120021-1 | Yongde, Yunnan | China | HZU |
| *Elsholtzia penduliflora* W.W.Sm. | Pan Li, PNLI20120037 | Kuming Botanical Garden | China | HZU |
| *Elsholtzia pilosa* (Benth.) Benth. | Pan Li, PNLI20120201-1 | Yajiang, Sichuan | China | HZU |
| *Elsholtzia rugulosa* Hemsl. | Pan Li, PNLI20120005-1 | Anning, Yunnan | China | HZU |
| *Elsholtzia saxatilis* (Kom.) Nakai ex Kitag. | Pan Li, PNLI20120060 | Shangzhi, Heilongjiang | China | HZU |
| *Elsholtzia souliei* H.Lév. | Pan Li, PNLI20120148 | Maerkang, Sichuan | China | HZU |
| *Elsholtzia splendens* Nakai ex F.Maek. | Pan Li, PNLI20120059 | Shangzhi, Heilongjiang | China | HZU |

| | | | | |
|---|---|---|---|---|
| *Elsholtzia splendens* Nakai ex F.Maek. | Joongku Lee, PNLI20120476 | Geumsan, Sindaeri | South Korea | HZU |
| *Elsholtzia stachyodes* (Link) Raizada & H.O.Saxena | Pan Li, PNLI20120295-1 | Shangri-La, Yunnan | China | HZU |
| *Elsholtzia stauntonii* Benth. | Yongming Yuan, YGY2012-026① | Dangchang, Gansu | China | HZU |
| *Elsholtzia strobilifera* (Benth.) Benth. | Pan Li, PNLI20120340 | Ninglang, Yunnan | China | HZU |
| *Elsholtzia winitiana* Craib | Pan Li, PNLI20120029-1 | Yongde, Yunnan | China | HZU |
| *Elsholtzia* sp. nov. | Pan Li, PNLI20120191 | Yajiang, Sichuan | China | HZU |
| *Keiskea australis* C.Y.Wu & H.W.Li | Binjie Ge, TQ02438 | Ningde, Fujian | China | CSH |
| *Keiskea elsholtzioides* Merr. | Pan Li, PNLI20120430-1 | Songyang, Zhejiang | China | HZU |
| *Keiskea elsholtzioides* Merr. | Pan Li, PNLI20120458-1 | Fuzhou, Fujian | China | HZU |
| *Keiskea glandulosa* C.Y.Wu | Wuyishan Expedition 801502 | Guangze, Fujian | China | FNU |
| *Keiskea japonica* Miq. | Tetsuo Ohi-Toma, PNLI20120049-1 | Koishikawa Botanical Garden | Japan | HZU |
| *Keiskea japonica* Miq. | Tetsuo Ohi-Toma, PNLI20120049-2 | Koishikawa Botanical Garden | Japan | HZU |
| *Keiskea macrobracteata* Masam. | Shih Wen Chung 3244 | Hualian, Taiwan | China | TAIF |
| *Mosla cavaleriei* H.Lév. | Pan Li, PNLI20120445 | Cangnan, Zhejiang | China | HZU |
| *Mosla chinensis* Maxim. | Pan Li, PNLI20120245 | Hangzhou, Zhejiang | China | HZU |
| *Mosla dianthera* (Buch.-Ham. ex Roxb.) Maxim. | Pan Li, PNLI20120248 | Ningguo, Anhui | China | HZU |
| *Mosla hangchowensis* Matsuda | Pan Li, PNLI20120424-1 | Yongkang, Zhejiang | China | HZU |
| *Mosla japonica* (Benth. ex Oliv.) Maxim. | Tadashi Minamitani, PNLI20120416 | Miyazaki Pref. | Japan | HZU |
| *Mosla scabra* (Thunb.) C.Y.Wu & H.W.Li | Pan Li, PNLI20120427 | Songyang, Zhejiang | China | HZU |
| *Mosla soochowensis* Matsuda | Pan Li, PNLI20120414 | Hangzhou, Zhejiang | China | HZU |
| *Mosla tamdaoensis* Phuong | Zhuozhou, C-K-393 | Tam Dao | Vietnam | KUN |
| *Perilla frutescens* (L.) Britton | Pan Li, PNLI20120096 | Wenchuan, Sichaun | China | HZU |
| *Perilla frutescens* var. *auriculato-dentata* C.Y.Wu & S.J.Hsuan ex H.W.Li | Pan Li, PNLI20130589 | Jinzhai, Anhui | China | HZU |
| *Perillula reptans* Maxim. | Tadashi Minamitani, PNLI20120417-1 | Miyazaki Pref. | Japan | HZU |
| *Perillula reptans* Maxim. | Tadashi Minamitani, PNLI20120417-2 | Miyazaki Pref. | Japan | HZU |
